# Uncovering accurate prognostic markers for high‐risk uveal melanoma through DNA methylation profiling

**DOI:** 10.1002/ctm2.1317

**Published:** 2023-07-21

**Authors:** Andrea Soltysova, Dana Dvorska, Viera Horvathova Kajabova, Martina Pecimonova, Klaudia Cepcekova, Andrej Ficek, Lucia Demkova, Verona Buocikova, Pavel Babal, Ivan Juras, Katarina Janikova, Ivana Kasubova, Marek Samec, Dusan Brany, Darina Lyskova, Jela Valaskova, Zuzana Dankova, Bozena Smolkova, Alena Furdova

**Affiliations:** ^1^ Department of Molecular Biology Faculty of Natural Sciences, Comenius University in Bratislava Bratislava Slovakia; ^2^ Institute for Clinical and Translational Research Biomedical Research Center, Slovak Academy of Sciences Bratislava Slovakia; ^3^ Biomedical Centre Martin, Jessenius Faculty of Medicine in Martin Comenius University in Bratislava Martin Slovakia; ^4^ Department of Molecular Oncology Cancer Research Institute, Biomedical Research Center of the Slovak Academy of Sciences Bratislava Slovakia; ^5^ Department of Pathology Faculty of Medicine, Comenius University in Bratislava Bratislava Slovakia; ^6^ Lambda Life a.s. Bratislava Slovakia; ^7^ Department of Pathophysiology, Jessenius Faculty of Medicine in Martin Comenius University in Bratislava Martin Slovakia; ^8^ Department of Ophthalmology Faculty of Medicine, Comenius University in Bratislava Bratislava Slovakia

DEAR EDITOR

Uveal melanoma (UM) is a rare, aggressive cancer with limited treatment options. Despite significant advancements in understanding its genetic background,^1^, ^2^ the precise contribution of epigenomic alterations to the pathogenesis and progression of the disease remain elusive. In this study, we utilized a carefully curated set of UM samples to define the epigenomic and transcriptomic landscapes of high‐risk tumours and identify novel, clinically relevant methylation markers and therapeutic targets.

We stratified UM patients into risk groups based on UM‐specific chromosomal rearrangements, particularly monosomy 3 (M3) and *BAP1* mutations in tumour tissues (Figure 1A). Please refer to the Supporting information for a detailed description of patient clinical characteristics and methods (Additional file 1: Tables S1‐S3). Specifically, we identified 25 low‐risk and 33 high‐risk patients, of which 21 (63.6%) carried the *BAP1* mutation (Figure 1B, C).

**FIGURE 1:**
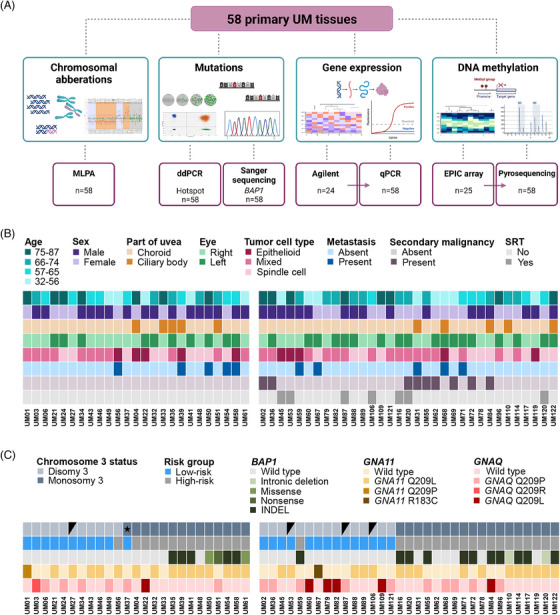
Baseline characteristics. (A) Overview of the methodological approaches performed in 58 samples, 25 subjected to whole‐genome analyses. (B) Clinico‐pathological characteristics of the included patients and (C) tumour mutational and chromosomal aberration status. On the left side are characterized samples analysed by whole‐genome approaches. Four samples marked with black triangles did not carry UM‐specific chromosomal aberrations. An asterisk highlights a sample with partial loss of chromosome 3. The high‐risk group was determined based on monosomy 3 or *BAP1* mutations in tumour tissue and refined by gene expression profiling. Insertions, deletions and one insertion/deletion mutation in the *BAP1* gene were combined as INDELs. SRT, stereotactic radiotherapy in the past.

We observed extensive gene expression reprogramming in high‐risk UMs, resulting in 2262 differentially expressed genes (DEGs) including 60 epigenetic regulators, histone modifiers and chromatin remodelers. Furthermore, we identified 44 398 differentially methylated CpGs, with hypermethylation more frequent in TSS1500 and CpG shores (Supporting Information Additional file 1: Figures S1, S2; Additional file 2: Tables S1‐4). Integrative analysis revealed 635 hypomethylated upregulated and 309 hypermethylated downregulated genes in high‐risk tumours (Figure 2A,B). A significant proportion of methylation‐regulated DEGs belong to specific functional groups, including epigenetic modifiers, transcription factors, tumour suppressor genes and oncogenes (Figure 2C), demonstrating the critical role of DNA methylation in controlling cell fate. The median β values of differentially methylated CpGs, were lower in the high‐risk UMs, suggesting that epigenetic gene activation can be more common than repression (Figure 2D). *BAP1* expression negatively correlated with cg01493712 DNA methylation β value (*r* = −.496; *p* = .014), implying epigenetic control of *BAP1* itself (Figure 2E). Aberrant DNA methylation, distributed relatively uniformly across the entire genome, was associated with the dysregulation of key oncogenic pathways such as EGFR tyrosine kinase inhibitor resistance, focal adhesion, proteoglycans in cancer, PI3K‐Akt signalling, or ECM‐receptor interaction (Figure 2F,G; Supporting Information Additional file 2: Table S5). These findings highlight the critical role of DNA methylation aberrancy in driving transcriptomic changes associated with poor prognosis.

**FIGURE 2:**
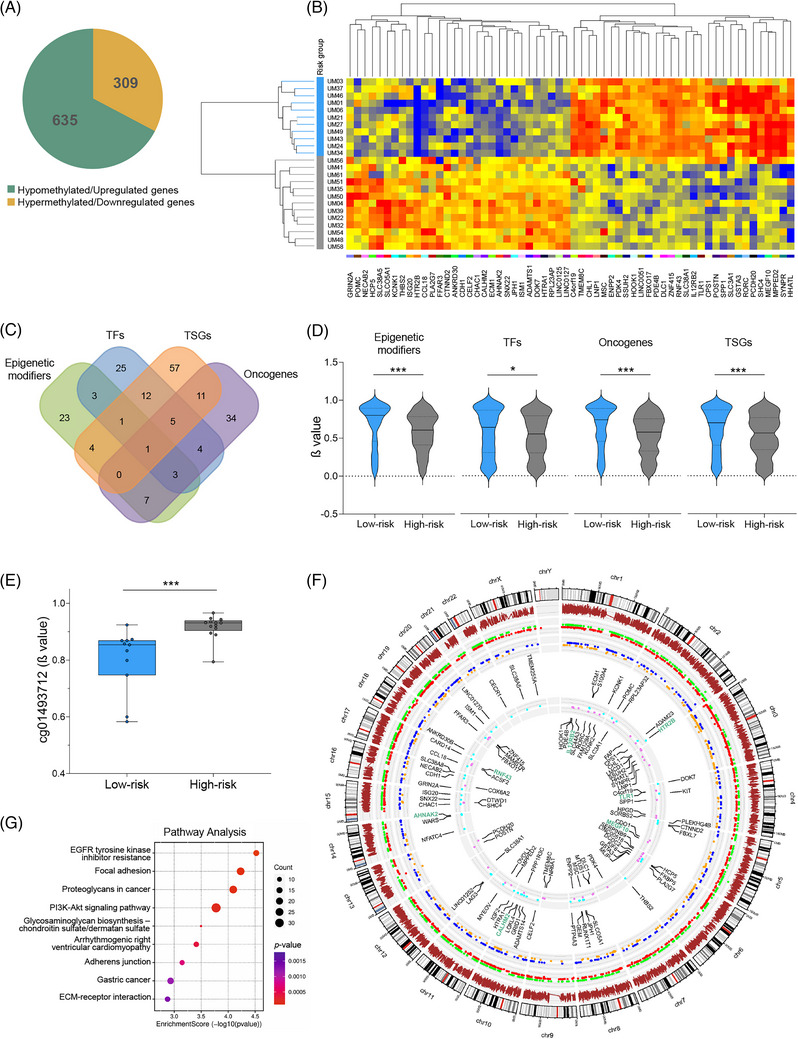
The integrated DNA methylation and gene expression analysis findings in 25 UM tissues. (A) Pie chart showing the proportion of hypomethylated upregulated and hypermethylated downregulated genes in high‐ versus low‐risk samples. (B) Heat map displaying gene expression of the top 30 DNA methylation‐regulated genes. (C) Venn diagram illustrating the overlap between individual functional groups in the number of DNA methylation‐regulated genes. (D) Violin plots comparing the quantitative differences in β values between high‐ and low‐risk tumours in individual functional groups. Differentially methylated CpGs (FDR adjusted *p*‐value < .05 with no threshold for β‐values) in DEGs were included only. (E) Box plot depicting the differences between high‐ and low‐risk samples in *BAP1* cg01493712 methylation β values. (F) Circos plot illustrating the relationship between individual gene expression and DNA methylation across the genome. Brown bars represent the culmination of data with Δβ > .15; green dots indicate upregulated genes, red dots represent downregulated genes, blue dots indicate hypomethylated upregulated genes and orange dots represent hypermethylateddownregulated genes. The inner circles list 50 upregulated (cyan) and downregulated genes (violet) with relevant CpG methylation. G) Pathway analysis for DNA methylation‐regulated genes. TFs, transcription factors; TSGs, tumour suppressor genes.

Based on integrative analysis findings, we selected nine candidate genes, three upregulated and six downregulated. The selection was guided by fold change (FC), Δβ values and the number of CpGs inversely correlated with gene expression. The upregulated genes were *HTR2B* (FC = 191.4), *AHNAK2* (FC = 12.6) and *CALHM2* (FC = 7.8), while the downregulated genes were *SLC25A38* (FC = −4.6), *EDNRB* (FC = −4.7), *TLR1* (FC = −8.6), *RNF43* (FC = −10.8), *IL12RB2* (FC = −18.1) and *MEGF10* (FC = −25.2). Their expression (Supporting Information Additional file 1: Figure S3) was significantly associated with UM overall survival (OS) data, available in The Cancer Genome Atlas dataset (Supporting Information Additional file 1: Figure S4).^3^ The correlation between DNA methylation percentage measured by pyrosequencing in 58 UM tumours and β values were highly significant (Supporting Information Additional file 1: Table S4). In addition, individual DNA methylation values showed minimal overlap between high‐ and low‐risk tissues (*p* < .001) (Figure 3A), indicating the potential use of these markers for predicting risk groups with excellent diagnostic accuracy. AUC values ranged from .870 to .956 (*p* < .001) (Figure 3B; Supporting Information Additional file 1: Table S5). By combining methylation values of hypomethylated *AHNAK2* and *CALHM2* genes with values of hypermethylated *IL12RB2* (Signature 1) or *SLC25A38* (Signature 2) genes, we achieved AUC values of .999 and .994, respectively (*p* < .001), demonstrating the robustness and potential clinical utility of these epigenetic markers in UM risk stratification. Kaplan–Meier survival curves were generated with the log‐rank test, and univariate Cox regression analysis was performed to confirm that DNA methylation of *CALHM2* and *MEGF10* genes and both methylation signatures were sufficient to stratify patients reasonably well as the standard risk groups based on chromosomal rearrangements and mutation profiling (Figure 3C, Table 1).

**FIGURE 3:**
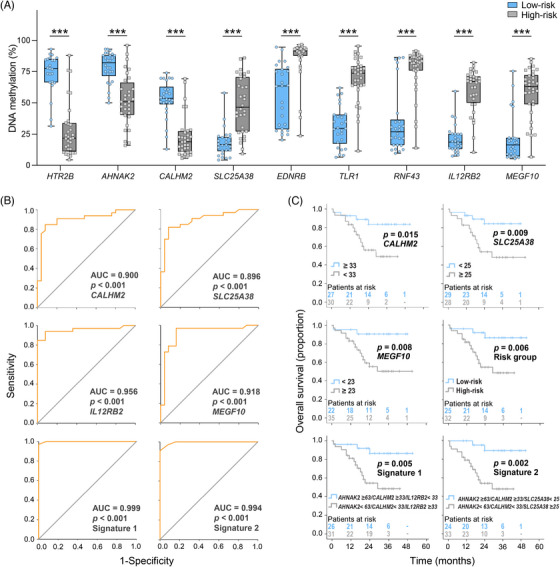
The prognostic performance of DNA methylation. (A) Differences in % of DNA methylation between high‐ and low‐risk tissues. (B) ROC curves with AUC and *p*‐values for selected genes and two methylation signatures. (C) Kaplan–Meier survival plots and log‐rank test *p*‐values for selected genes, risk group and two methylation gene signatures, Signature 1: *AHNAK2, CALHM2* and *IL12RB2*; Signature 2: *AHNAK2, CALHM2* and *SLC25A38*. Cut‐off values representing % of DNA methylation were selected based on the Youden index. ROC, receiver operating characteristic curve; AUC, area under the curve.

**TABLE 1:** Univariate and multivariate Cox regression models for clinical variables and DNA methylation of all selected genes.

	Univariate			Multivariate*		
Variables	***p*‐value**	**HR**	**95.0% CI**	***p*‐value**	**HR**	**95.0% CI**
Age	.264	.98	.94–1.02			
Volume (cm^3^)	**.002**	2.28	1.36–3.85	**.008**	2.00	1.20–3.35
Dg. C69.4	**.001**	3.77	1.67–8.52	**.026**	2.67	1.13–6.31
Epithelial & mixed cell type	**.023**	3.70	1.20–11.38			
Ki‐67 (>10%)	**.030**	3.01	1.11–8.16	**.033**	3.07	1.10–8.60
Metastasis	**.021**	3.24	1.19–8.78			
Secondary malignancy	.395	.53	.12–2.31			
High‐risk group^&^	**.014**	4.83	1.38–16.89	**.019**	4.57	1.28–16.29
*HTR2B <* 61%	.058	2.97	.97–9.14			
*AHNAK <* 63%	.086	2.40	.89–6.49			
*CALHM2 <* 33%	**.025**	3.62	1.18–11.15	**.018**	4.38	1.29–14.88
*SLC25A38 ≥* 25%	**.016**	3.99	1.30–12.25			
*EDNRB ≥* 84%	.338	1.60	.61–4.22			
*TLR1 ≥* 59%	.174	1.96	.74–5.16	**.036**	3.43	1.08–10.87
*RNF43* ≥ 45%	.096	2.59	.84–7.96			
*IL12RB2* ≥ 34%	.063	2.90	.95–8.92			
*MEGF10* ≥ 23%	**.019**	5.85	1.34–25.67	**.024**	5.59	1.26–24.82
*Signature 1*	**.012**	4.95	1.42–17.31	**.018**	4.61	1.30–16.41
*Signature 2*	**.009**	7.27	1.66–31.92	**.011**	7.03	1.58–31.33

* Clinical variables with *p* < .1 from univariate analysis were included in multivariate models; only values for significant variables are presented.

^&^ Defined based on UM‐specific chromosomal rearrangements and *BAP1* mutations. Cut‐off values for individual genes, representing % of DNA methylation, were selected based on the Youden index. Signature 1: *AHNAK2*, *CALHM2* *and IL12RB*; Signature 2: *AHNAK2* *CALHM2* and *SLC25A38*.

The DNA methylation repatterning in UM was initially attributed to the loss of *BAP1*, a gene coding for a deubiquitinating hydrolase that exerts diverse functions such as cell cycle regulation, DNA damage repair, chromatin remodelling and gene expression control.^4^ Although UM is considered poorly immunogenic due to its immune‐privileged site of origin, it has been proposed that BAP1 loss may promote the immunosuppressive tumour microenvironment (TME).^5^ UM is a unique tumour type in which a high density of tumour‐infiltrating lymphocytes and tumour‐associated macrophages paradoxically correlates with a worse prognosis, highlighting the complex interaction between the TME and the immune response. Epigenetic regulations play a critical role in shaping these intricate relations.^6^ Accordingly, six of the top nine methylation‐regulated genes have been previously linked to immune functions. Specifically, *EDNRB, IL12RB2, CALHM2* and *RNF43*, were identified among UM prognostic genes that interact with immune and stromal cells in the TME.^7^ IL‐12Rβ2, a subunit of the IL‐12 receptor, generates high‐affinity binding sites for IL‐12, one of the most potent antitumor cytokines.^8^ The prognostic significance of *CALHM2* and *RNF43* is further reinforced by their listing among the most important DEGs related to UM survival.^3^ Additionally, *AHNAK2*, shown to promote UM cell proliferation and migration,^9^ was found to correlate with infiltration of immune cell subpopulations such as CD8^+^ and CD4^+^.^10^ Our findings align with a recent report by Figueiredo et al.,^5^ which revealed that the downregulation of *TLR1*, a gene responsible for immune activation, is correlated with M3 status but not *BAP1* expression, indicating epigenomic reprogramming independent of *BAP1* mutations. These results highlight the importance of further exploring epigenetic regulation of the unique immune landscape of UM, which presents both challenges and opportunities for developing effective treatments for high‐risk patients.

Overall, our study provides compelling evidence for the substantial role of DNA methylation in UM progression by regulating the expression of genes involved in critical biological processes such as immune evasion, calcium homeostasis, adhesion and migration. Importantly, we demonstrate that the DNA methylation status of carefully selected CpG sites has the potential to serve as reliable prognostic biomarkers, underscoring the clinical relevance of DNA methylation analysis in UM. By leveraging the power of epigenetic profiling, we can gain a powerful tool for patient stratification, which can aid in personalized therapy and ultimately lead to improved outcomes.

## CONFLICT OF INTEREST STATEMENT

The authors declare that they have no competing interests.

## FUNDING INFOMATION

This research was funded by the Slovak Research and Development Agency Grant number APVV‐17‐0369, The Ministry of Education, Science, Research and Sport of the Slovak Republic, Grant number VEGA 1/0395/21 and LISPER (ITMS 313011V446: Integrative strategy in the development of personalized medicine of selected malignant tumors and its impact on quality of life. Operational program integrated infrastructure 2014−2020) projects.

## Supporting information

Supporting Inormation

Supporting Inormation

**Figure S1**. Transcriptomic profile of high‐risk compared to low‐risk tumors.

**Figure S2**. Differences in DNA methylation between high‐ and low‐risk uveal melanomas.

**Figure S3**. Validation of mRNA expression by qPCR.

**Figure S4**. Kaplan‒Meier survival plots constructed based on the gene expression of selected genes from The Cancer Genome Atlas (TCGA) and the Genotype‐Tissue Expression (GTEx) databases using the online Gene Expression Profiling Interactive Analysis (GEPIA) tool.
