# Supplementary material for: Uncovering accurate prognostic markers for high‐risk uveal melanoma through DNA methylation profiling
Source: Clin Transl Med. 2023 Jul 21;13(7):e1317. doi: 10.1002/ctm2.1317 (PMC10361544; doi:10.1002/ctm2.1317)
Supplement: Supplementary file 1 — Supporting Inormation [file CTM2-13-e1317-s001.docx]

**Additional File 1**

**Materials and methods**

**Patient samples**

This study involved 58 UM patients treated by enucleation (7 of whom were enucleated after stereotactic radiosurgery in the past) between August 2018 and January 2022 at the Department of Ophthalmology, Faculty of Medicine, Comenius University in Bratislava. The median age was 66 years (ranging from 32 to 87), the right and left eyes were affected approximately equally (51.7%, n = 30 vs. 48.3%, n = 28), and 58.6% (n = 34) of patients were males. Choroidal melanoma (C69.3) was diagnosed in 82.8% (n = 48) of cases, while ciliary body melanoma (C69.4) was diagnosed in 17.2% (n = 10) of cases. All UM tissues underwent routine histological examination at the Department of Pathology, Faculty of Medicine, Comenius University in Bratislava. Ten patients were diagnosed with stage IV disease, and one developed metastasis after primary UM treatment. Metastases were located in the liver (n = 4), lungs (n = 3), spine (n = 1), skin (n = 2), and pelvis (n = 1).

**Patient stratification**

Based on multiplex ligation-dependent probe amplification (MLPA) results, 32 samples were classified as monosomy (M3) and 22 as disomy 3 (D3). Three of four samples that did not exhibit any UM-specific rearrangements were previously treated by stereotactic radiotherapy. The classification of two samples, UM37 and UM56, was further refined by gene expression profiling (GEP). UM37 exhibited only partial loss of chromosome 3 (3q-6p+8q+), and UM56 showed loss of the short arm of chromosome 1 (1p-8q+) without any aberrations associated with chromosome 3. Based on the expression of 12 discriminatory genes^1^ extracted from gene expression data, UM37 was classified as low-risk and UM56 as high-risk UM using unsupervised clustering (Figure S1A). Additionally, UM59, the only D3 sample carrying *BAP1* mutation, was considered high-risk.

**Genomic profiling**

Tumour samples (approx. 0.5 cm^2^) snap-frozen in liquid nitrogen after surgery and stored at -80°C were mechanically homogenized, and DNA was extracted by a QIAamp DNA Mini Kit (Qiagen, Hilden, Germany). Isolated DNA was used for genomic and methylome profiling. DNA quantity and quality were evaluated by a Nanodrop 2000 spectrophotometer (NanoDrop Technologies, USA).

**Chromosomal rearrangements**

Chromosomal aberrations were assessed in 100 ng of tumour DNA by MLPA using the SALSA MLPA Probemix P027 Uveal melanoma kit (MRC Holland, the Netherlands) ^1^. MLPA products were separated by capillary electrophoresis on a Genetic Analyzer 3130XL (Applied Biosystems, MA, USA) and analyzed by Coffalyser software (MRC Holland, the Netherlands).

**Hotspot mutations**

Nine hotspot mutations in four genes, GNAQ (p. Q209L, p. Q209R, p. Q209P, p. R183Q), GNA11 (p. Q209L, p. Q209P, p. R183C), PLCB4 (p. D630Y) and CYSLTR2 (p. L129Q) were determined in tumour tissues by digital droplet PCR (ddPCR) and validated by Sanger sequencing as described previously ^2^.

**BAP1 mutations**

All 17 coding exons of the *BAP1* gene (ENSG00000163930) were amplified using HOT FIREPol® DNA Polymerase (Solis BioDyne, Estonia) with 10 μM specific primers (Additional file 1: Table S1) and the following PCR steps: denaturation at 95°C for 15 min; 30 cycles at 95°C for 40 s, 60°C for 30 s, and 72°C for 60 s; and a final 20 min at 72°C. PCR products were purified with Illustra™ ExoProStar 1-Step enzyme (Sigma Aldrich, USA), amplified using the BigDye™ Terminator v3.1 Cycle Sequencing Kit (Applied Biosystems, USA), and purified according to the manufacturer's protocol. Sequencing products were analyzed on a Genetic Analyzer 3130XL (Applied Biosystems, MA, USA). ChromasPro Software v1.6 (Technelysium Pty Ltd, Australia) was applied to identify variants by alignment to the reference sequence (ENST00000460680.6).

**Transcriptome profiling and validation**

Total RNA was isolated from snap-frozen tumour tissues by an RNeasy Mini Kit (Qiagen, Hilden, Germany). The RNA quantity and quality were evaluated on an Agilent 2100 Bioanalyzer instrument using an Agilent RNA 6000 Nano Kit (Agilent Technologies, USA). Only samples with RIN above 8.5 were selected for transcriptome analysis, while samples with RIN above 7.5 were rated suitable for qPCR.

Agilent SurePrint G3 Human Gene Expression 8x60K v3 Microarray was used to obtain transcriptomic data from 24 tumour samples (one sample was excluded due to insufficient RNA quality). After the integrity check, 100 ng of total RNA was labelled with a Low Input Quick Amp Labeling Kit (Agilent, Santa Clara, CA, USA). Labelled targets were repurified using the GeneJET^TM^ RNA Purification Kit (Thermo Scientific, USA). Samples with specific activity above eight were fragmented and prepared for hybridization using a Gene Expression Hybridization Kit (Agilent Technologies, USA), applied onto the array, and hybridized for 17 hours at 65°C. Microarray slides were washed in two steps using a Gene Expression Wash Buffer Kit (Agilent Technologies, USA) and scanned by a SureScan Microarray Scanner (Agilent Technologies, USA) at a resolution of 2 µm.

Automatic image processing was performed with Feature Extraction Software 12.0.3.2 (Agilent Technologies, USA). The resulting files with spot intensities were imported into GeneSpring 14.9 GX software (Agilent Technologies, USA). Differential gene expression between high- vs. low-risk samples was calculated using the moderated t-test and Benjamini‒Hochberg correction with abs(FC) ≥ 2 and FDR adjusted *p*-value ˂ 0.05 set as cut-offs. The g:profiler toolkit was used for gene set enrichment analysis ^3^.

**Quantitative RT‒PCR**

The top nine selected genes were validated by qPCR using the primers listed in Additional file 1: Table S2. Total RNA was reverse transcribed with RevertAid First Strand cDNA Synthesis Kit (Thermo Fisher Scientific, USA). The 20 µl qPCRs performed in triplicate contained 0.3 µM primers, 50 ng of RNA, and GoTaq® qPCR Master Mix (Promega, USA). qPCR was carried out on a Stratagene Mx3005P Real-time PCR System (Agilent Technologies, USA) under the following thermal cycling conditions: 95°C for 10 min, followed by 36 cycles of 95°C for 20 s, 59°C for 20 s, and 72°C for 20 s. Relative gene expression was quantified using the 2^–ΔΔCT^ method. The results were reported as fold change (FC) in the high-risk group normalized to endogenous control *HPRT1* and relative to the low-risk group.

**Methylome profiling and validation**

DNA isolated from 25 UM tissues was bisulfite-treated using the EZ DNA Methylation™ Kit (Zymo Research, USA) according to Illumina's recommended deamination protocol. Genome-wide methylation screening using an Illumina Infinium Methylation EPIC array BeadChip (850K) (Illumina, San Diego, CA, USA) was carried out by Epigenomic Services from Diagenode. The raw data were checked for bisulfite conversion efficiency, staining, extension, hybridization, target removal, and dye specificity. Arrays that passed quality control were processed via a pipeline included in the R ChAMP package ^4^ with default settings except XY filtering, as males and females were evenly distributed. The batch effect was corrected through the ComBat algorithm ^5^. For each CpG site, the individual methylation beta value (β) was acquired, ranging from 0 for unmethylated to 1 for fully methylated. Differentially methylated CpGs were obtained based on an FDR-adjusted *p* value < 0.05 with no threshold set for β values.

**Integration of whole-genome methylation and gene expression data**

Integrative analysis of transcriptomic and epigenomic data was performed to identify methylation-driven transcriptomic changes. Pearson or Spearman correlation coefficients were calculated based on data distribution, and genes with a significant negative correlation between gene expression and individual β values were considered DNA methylation-regulated. Epigenetic regulators, their complexes, targets, and products were identified based on the EpiFactors database ^6^, and TSGs and oncogenes were identified by the TSGene 2.0 and ONGene databases ^7,8^.

**Pyrosequencing**

DNA methylation of prioritized loci located within regulatory regions of nine selected genes was validated by pyrosequencing. Primer design was guided by the whole-genome data, focusing on the largest Δβ values (difference between high- and low-risk tumours). Primers for PCR amplification and pyrosequencing were designed using PyroMark Assay Design Software 2.0 (Qiagen, Hilden, Germany), and their sequences are listed in Additional file 1: Table S3. The PyroMark PCR Kit*^®^* (Qiagen, Hilden, Germany) was used for PCR amplification of bisulfite-converted DNA (EpiTect Bisulfite Kit*^®^* (Qiagen, Hilden, Germany)). The concentrations of PCR primers were 0.32 μM. The annealing temperature was 57°C for all genes except for *CALHM2* and *AHNAK2*, where the annealing temperature was 60°C. Pyrosequencing was carried out on the PyroMark Q96 ID device (Qiagen, Hilden, Germany) with the PyroGold Reagent Kit *^®^* (Qiagen, Hilden, Germany). The pyrosequencing results were analyzed by PyroMark Q96 Software version 2.5.8 (Qiagen, Hilden, Germany).

**Statistical analysis**

IBM SPSS Statistics for Windows, Version 23.0 (Armonk, NY: IBM Corp.) was used for statistical analysis. The normality of data distribution was assessed by the Shapiro‒Wilk test, based on which the Mann‒Whitney U test or Student's t-test was applied to analyze differences between studied groups. All tests were two-tailed and performed at a significance level of α = 0.05. Categorical variables were tested using χ2 or Fisher's exact test. The Pearson or Spearman correlation coefficients were calculated based on the data distribution. DNA methylation values were evaluated for their performance in predicting the risk groups (defined by chromosome 3 or *BAP1* mutation status) by calculation of the area under the receiver operating characteristic (ROC) curve (AUC). Based on this analysis, the optimal DNA methylation cut-off values for individual genes were estimated from the Youden Index ^9^. Kaplan–Meier survival analysis with the log-rank test was used to assess the differences in overall survival (OS) based on these cut-offs. The variables with univariate Cox proportional hazard model *p*-values < 0.1 were considered in a multivariate model with backward selection. The formula by Gass was applied to calculate tumour volume: "TV = π/6 × (largest basal diameter × width × prominence)" ^10^.

**References:**

1. Onken MD, Worley LA, Ehlers JP, Harbour JW. Gene expression profiling in uveal melanoma reveals two molecular classes and predicts metastatic death. Cancer Res. Oct 15 2004;64(20):7205-9. doi:10.1158/0008-5472.Can-04-1750

2. Soltysova A, Sedlackova T, Dvorska D, et al. Monosomy 3 Influences Epithelial-Mesenchymal Transition Gene Expression in Uveal Melanoma Patients; Consequences for Liquid Biopsy. Int J Mol Sci. Dec 17 2020;21(24)doi:10.3390/ijms21249651

3. Raudvere U, Kolberg L, Kuzmin I, et al. g: Profiler: a web server for functional enrichment analysis and conversions of gene lists (2019 update). Nucleic acids research. 2019;47(W1):W191-W198.

4 Tian Y, Morris TJ, Webster AP, et al. ChAMP: updated methylation analysis pipeline for Illumina BeadChips. Bioinformatics. 2017;33(24):3982-3984. doi:10.1093/bioinformatics/btx513

5. Johnson WE, Li C, Rabinovic A. Adjusting batch effects in microarray expression data using empirical Bayes methods. Biostatistics. Jan 2007;8(1):118-27. doi:10.1093/biostatistics/kxj037

6. Medvedeva YA, Lennartsson A, Ehsani R, et al. EpiFactors: a comprehensive database of human epigenetic factors and complexes. Database (Oxford). 2015;2015:bav067. doi:10.1093/database/bav067

7. Zhao M, Kim P, Mitra R, Zhao J, Zhao Z. TSGene 2.0: an updated literature-based knowledgebase for tumor suppressor genes. Nucleic Acids Research. 2015;44(D1):D1023-D1031. doi:10.1093/nar/gkv1268

8. Liu Y, Sun J, Zhao M. ONGene: a literature-based database for human oncogenes. Journal of Genetics and Genomics. 2017;44(2):119-121.

9. Youden WJ. Index for rating diagnostic tests. Cancer. Jan 1950;3(1):32-5. doi:10.1002/1097-0142(1950)3:1<32::aid-cncr2820030106>3.0.co;2-3

10. Gass JD. Comparison of uveal melanoma growth rates with mitotic index and mortality. Arch Ophthalmol. Jul 1985;103(7):924-31. doi:10.1001/archopht.1985.01050070050028

**
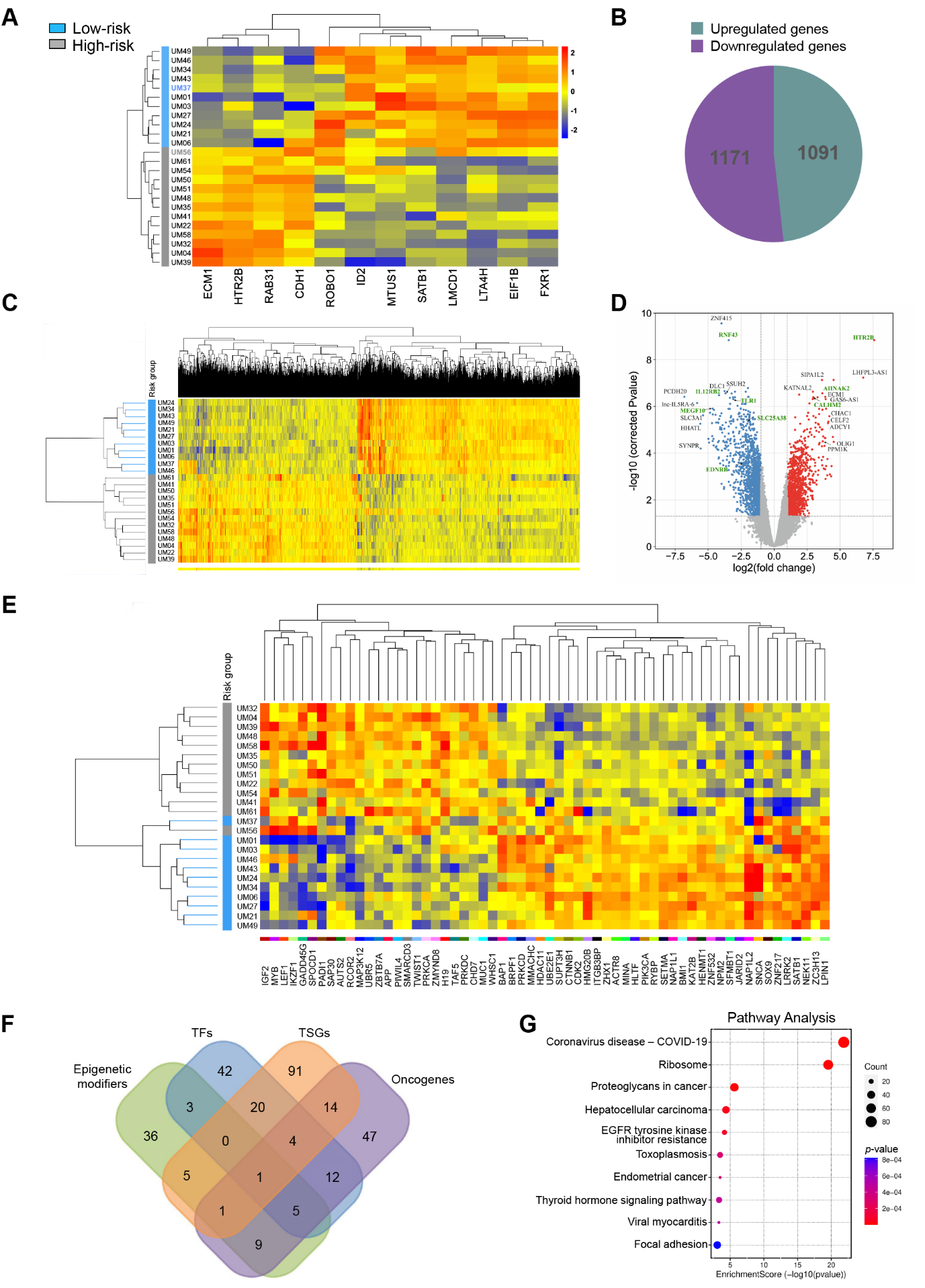
**

**Figure S1.** Transcriptomic profile of high-risk compared to low-risk tumours. A) Heatmap of 12-gene discriminatory signature expression. Light blue highlights the low-risk cluster, and grey highlights the high-risk cluster. B) Pie diagram showing the total number of differentially expressed genes (DEGs). C) Heatmap of DEGs with abs(FC) ≥ 2 and FDR adjusted *p*-value < 0.05 between risk groups. D) Volcano scatter plot showing the distribution –log10(p-value) (y-axis) and log2(fold change) (x-axis) of mRNA expression in the high-risk vs. low-risk groups. Green highlighted genes were selected for validation. E) Heatmap of differentially expressed epigenetic regulators. F) Venn diagram demonstrating the overlap in DEGs between differentially expressed epigenetic modifiers, transcription factors, tumour suppressor genes, and oncogenes. G) Deregulated signalling pathways in high-risk tumours. Abbreviations: TFs, transcription factors; TSGs, tumour suppressor genes

**
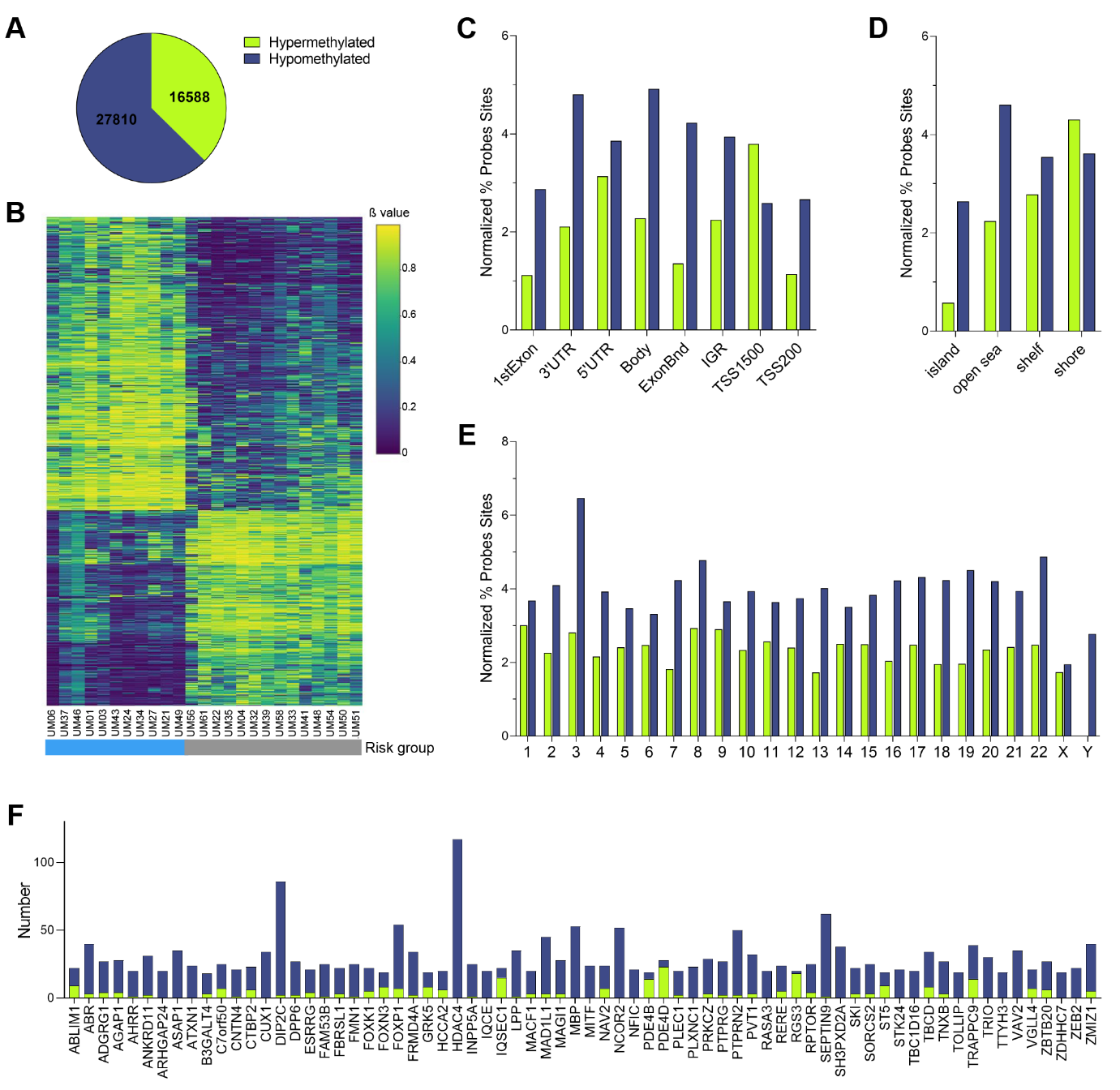
Figure S2.** Differences in DNA methylation between high- and low-risk uveal melanomas (UMs). A) The number of differentially methylated CpGs. B) Unsupervised clustering of the top 5,000 differentially methylated CpGs in UM tissues. Blue highlights are low-risk samples, and grey highlights are high-risk samples. C) Distribution of differentially methylated CpGs across the regulatory regions and D) CpG islands, normalized to the overall number of probes present within each of those regions contained in the Human Infinium Methylation EPIC Bead Chip array. E) Distribution of differentially methylated CpGs across the individual chromosomes, normalized to the overall number of probes present on the chip within each chromosome. F) Top 70 genes enriched by significant CpGs. Abbreviations: Bnd, boundaries; IGR, intergenic region; TSS, transcription start site





**Figure S3.** Validation of mRNA expression by qPCR. A) samples with transcriptomic data (n = 24), B) all samples (n=58); *MEGF10* was not expressed (Ct above 35) in high-risk samples when analyzed by qPCR.


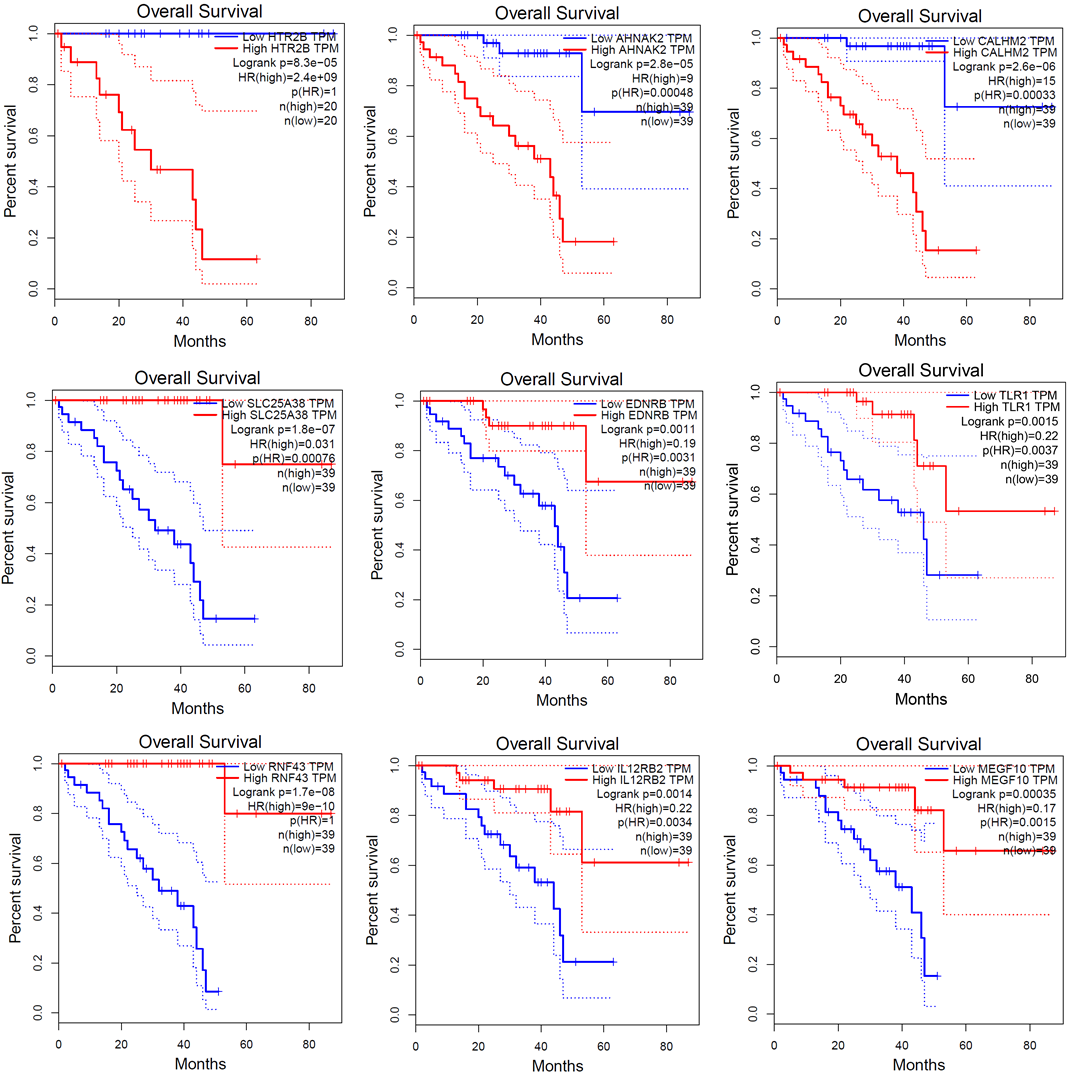
**Figure S4.** Kaplan‒Meier survival plots constructed based on the gene expression of selected genes from The Cancer Genome Atlas (TCGA) and the Genotype-Tissue Expression (GTEx) databases using the online Gene Expression Profiling Interactive Analysis (GEPIA) tool. Available online: <http://gepia.cancer-pku.cn/> (accessed on 16 December 2022).

**Table S1.** Sequencing primers used for *BAP1* mutation detection

| **Name** | **Strand** | **Sequence (5′-3′)** | **PCR product (bp)** |
| --- | --- | --- | --- |
| ***BAP1* ex1-3** | Forward | CGTTGTCTGTGTGTGGGACT | 504 |
|  | Reverse | CCCTGTTCTCTGGGACCTTC |  |
| ***BAP1* ex4-5** | Forward | GGATCACAGCAAGGACACCT | 850 |
|  | Reverse | TCATGTGGTAGCATTCCCAGT |  |
| ***BAP1* ex6-8** | Forward | TTTGCCTTCCACCCATAGTC | 779 |
|  | Reverse | ATCACCTGGATACTCTCTGTCC |  |
| ***BAP1* ex9-10** | Forward | GGGTGGGGCCTATACCTACA | 847 |
|  | Reverse | GAAGAACACTGCCCAAGGAC |  |
| ***BAP1* ex11-12** | Forward | TCTCTGGGAAGTGCTGGTTC | 1045 |
|  | Reverse | CAGGTGCTCAACATTATCTGCT |  |
| ***BAP1* ex13** | Forward | CTGGAGGTCGGGATGTATTT | 688 |
|  | Reverse | TGGGAAGAGAGGTCACAAGAA |  |
| ***BAP1* ex14-15** | Forward | AGGAGGGAGGAGGGAAGTG | 681 |
|  | Reverse | AGAGCACTGGAGCCAATCTT |  |
| ***BAP1* ex16-17** | Forward | TGATTTCTCCAGTTGCCTGA | 639 |
|  | Reverse | GAAGGAATGTGGCCTGGTT |  |

**Table S2.** Sequences of qPCR primers

| **Gene** | **Strand** | **Sequence (5′-3′)** | **PCR product (bp)** |
| --- | --- | --- | --- |
| *HTR2B* | Forward | GCTGATTTGCTGGTTGGATT | 134 |
|  | Reverse | ATGGATGCGGTTGAAAAGAG |  |
| *AHNAK2* | Forward | GGGGTCTTCACCTGTCTACG | 167 |
|  | Reverse | CCTGGACAGCCTCTGGACG |  |
| *CALHM2* | Forward | GCAAGGAGAACCCTGACAAC | 122 |
|  | Reverse | GCTTGAGGCACTTGGTCA |  |
| *SLC25A38* | Forward | CGCCCTTCTCAGGAATCTAC | 119 |
|  | Reverse | ACCAGCAAATATCCCACAGC |  |
| *EDNRB* | Forward | CGAATCTGCTTGCTTCATCC | 163 |
|  | Reverse | TGCCACTTTTCTTTCTCAACA |  |
| *TLR1* | Forward | TCCTCGTTAGAAGAAATCAGGA | 149 |
|  | Reverse | AATGGCAAAATGGAAGATGC |  |
| *RNF43* | Forward | GAGCCTGGATTCATCAGCAT | 123 |
|  | Reverse | GTCAAAGAGGACAGCACTGG |  |
| *IL12RB2* | Forward | TTGAGAGCACAAACACCAGAA | 170 |
|  | Reverse | GTCAGCTCCTGCAAGGTCAC |  |
| *MEGF10* | Forward | TTATGAAAGCGGGGAAATGT | 133 |
|  | Reverse | ATCACCATCGCAGGCACT |  |
| *HPRT1* | Forward | AAGTTCTTTGCTGACCTGCTG | 175 |
|  | Reverse | AGACGTTCTTTCCAGTTAAAGTTG |  |

**Table S3**. Sequences of pyrosequencing primers

| **Gene** | **Strand** | **Sequence**  **(5′-3′)** | **PCR product**  **(bp)** | **Number of CpG analyzed** |
| --- | --- | --- | --- | --- |
| *HTR2B* | Forward | AGTGTTTGGTATGGTTATAATGTTT |  |  |
|  | Reverse | Btn-ACACTCTATAAAAAAAAACCATTTACTATT | 127 | 2 |
|  | Sequencing | AGTTATTAATTATGTTGATTATTGT |  |  |
| *AHNAK2* | Forward | TGGGTTGGTTGTGTGATT |  |  |
|  | Reverse | Btn-TACCAAAACCCCCACATCC | 181 | 1 |
|  | Sequencing | GTTGGTTGTGTGATTTAGA |  |  |
| *CALHM2* | Forward | GGGGTTTTGGTTTTGAGTATTTT |  |  |
|  | Reverse | Btn-CCTACCCCCCATAACAATTT | 144 | 3 |
|  | Sequencing | TTGGTTTTGAGTATTTTGTAG |  |  |
| *SLC25A38* | Forward | TTGGGTAGATTAGGTTTGGATTGTA |  |  |
|  | Reverse | Btn-ACCTAATAAAAACCCCCAAAACTAAAAAAT | 132 | 3 |
|  | Sequencing | AGATTAGGTTTGGATTGTAAATA |  |  |
| *EDNRB* | Forward | TTGGAGGAAATTTATGGGGATAGAT |  |  |
|  | Reverse | Btn-ACTAAAACAAAATCAAAACCTTTTACAT | 95 | 3 |
|  | Sequencing | GGGATAGATTTTTATTTTTAGTTTA |  |  |
| *TLR1* | Forward | AGTTTGTATTGGTATTTGTGTATTAGTTT |  |  |
|  | Reverse | Btn-ACAACTACTCTAAAAAAAAACCAAAA | 169 | 2 |
|  | Sequencing | ATATTTTTTTGGGAATAATAT |  |  |
| *RNF43* | Forward | AGTGTTTAATTTAGTTTTAGGGGATAGT |  |  |
|  | Reverse | Btn-CAAACCAAATACCCCATACTACA | 131 | 3 |
|  | Sequencing | AGTTTTAGGGGATAGTATT |  |  |
| *IL12RB2* | Forward | AATGGAAGTTTTAGTATGTTTTGTT |  |  |
|  | Reverse | Btn-AAAACTTTCCTCCCAAATATCCTTTCTCTA | 139 | 4 |
|  | Sequencing | ATGTTTTGTTAGTATTTTAGTGGG |  |  |
| *MEGF10* | Forward | TGGTTGGTTTTGGGTGAAG |  |  |
|  | Reverse | Btn-ATAACCCCCCCCCATAAAAAACCTTC | 147 | 5 |
|  | Sequencing | GGTTTTGGGTGAAGAT |  |  |

**Table S4.** Correlation between methylation beta values for individual probes and % of DNA methylation obtained by analysis of identical CpGs by pyrosequencing.

| **Gene** |  | **n** | **r** | ***p*-value** |
| --- | --- | --- | --- | --- |
| *HTR2B* | cg22203451 | 23 | 0.898 | < 0.001 |
|  | cg19509703 | 23 | 0.881 | < 0.001 |
| *AHNAK2* | cg01513078 | 24 | 0.968 | < 0.001 |
| *CALHM2* | cg21686808 | 24 | 0.875 | < 0.001 |
|  | cg23175074 | 24 | 0.794 | < 0.001 |
|  | cg09336982 | 24 | 0.868 | < 0.001 |
| *SLC25A38* | cg23850760 | 24 | 0.745 | < 0.001 |
|  | cg08206536 | 24 | 0.675 | < 0.001 |
| *EDNRB* | cg13866767 | 24 | 0.879 | < 0.001 |
| *TLR1* | cg22809983 | 24 | 0.717 | < 0.001 |
|  | cg00646813 | 24 | 0.722 | < 0.001 |
| *RNF43* | cg09120724 | 24 | 0.736 | < 0.001 |
|  | cg22223182 | 24 | 0.553 | 0.005 |
| *IL12RB2* | cg19745415 | 24 | 0.885 | < 0.001 |
|  | cg00318756 | 24 | 0.848 | < 0.001 |
|  | cg06952660 | 24 | 0.851 | < 0.001 |
| *MEGF10* | cg26465611 | 24 | 0.876 | < 0.001 |

Abbreviations: r, correlation coefficient

**Table S5.** Individual AUC values calculated from the mean methylation values of all CpGs analyzed for individual genes obtained by pyrosequencing and two methylation signatures.

| **Gene** | **AUC**  n=58 | ***p*-value** | **AUC**  n=25* | ***p*-value** | **AUC**  n=33^&^ | ***p*-value** |
| --- | --- | --- | --- | --- | --- | --- |
| *HTR2B* | 0.935 | < 0.001 | 0.929 | < 0.001 | 0.939 | < 0.001 |
| *AHNAK2* | 0.870 | < 0.001 | 0.890 | 0.001 | 0.861 | < 0.001 |
| *CALHM2* | 0.900 | < 0.001 | 0.974 | < 0.001 | 0.852 | 0.001 |
| *SLC25A38* | 0.896 | < 0.001 | 0.961 | < 0.001 | 0.867 | < 0.001 |
| *EDNRB* | 0.898 | < 0.001 | 0.942 | < 0.001 | 0.872 | < 0.001 |
| *TLR1* | 0.942 | < 0.001 | 0.929 | < 0.001 | 0.953 | < 0.001 |
| *RNF43* | 0.887 | < 0.001 | 0.925 | < 0.001 | 0.861 | < 0.001 |
| *IL12RB2* | 0.956 | < 0.001 | 1.000 | < 0.001 | 0.930 | < 0.001 |
| *MEGF10* | 0.918 | < 0.001 | 0.922 | < 0.001 | 0.921 | < 0.001 |
| *Signature 1* | 0.999 | < 0.001 | 1.000 | < 0.001 | 1.000 | < 0.001 |
| *Signature 2* | 0.994 | < 0.001 | 1.000 | < 0.001 | 0.985 | < 0.001 |

***** samples analyzed by whole genome approaches; ^&^ samples not included in the whole genome dataset; n=58, all samples
