# Supplementary figures and images for: Uncovering accurate prognostic markers for high‐risk uveal melanoma through DNA methylation profiling
Source: Clin Transl Med. 2023 Jul 21;13(7):e1317. doi: 10.1002/ctm2.1317 (PMC10361544; doi:10.1002/ctm2.1317)

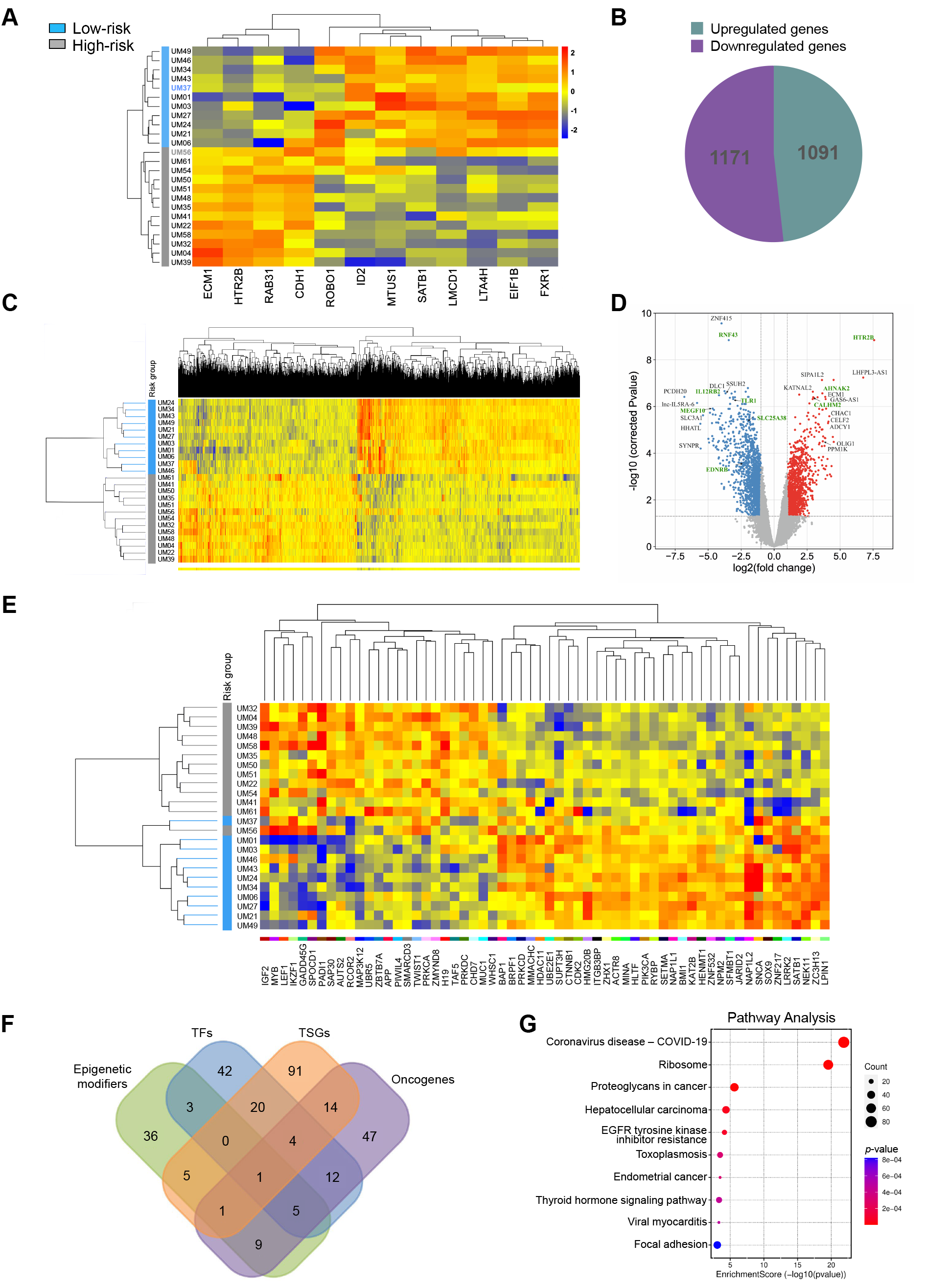

Supplement: Supplementary file 3 — Figure S1. Transcriptomic profile of high‐risk compared to low‐risk tumors. [file CTM2-13-e1317-s004.tif]

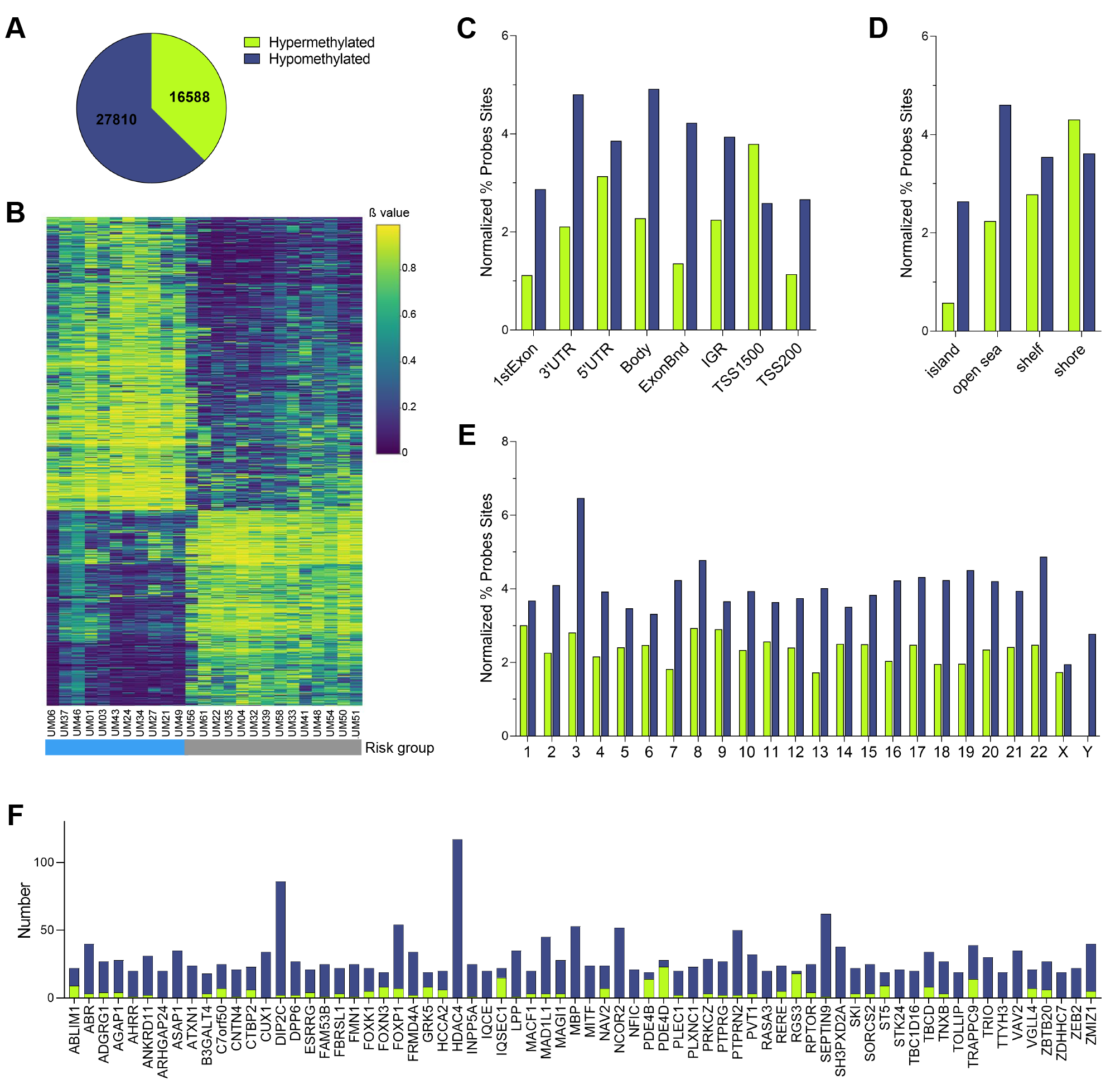

Supplement: Supplementary file 4 — Figure S2. Differences in DNA methylation between high‐ and low‐risk uveal melanomas. [file CTM2-13-e1317-s006.tif]

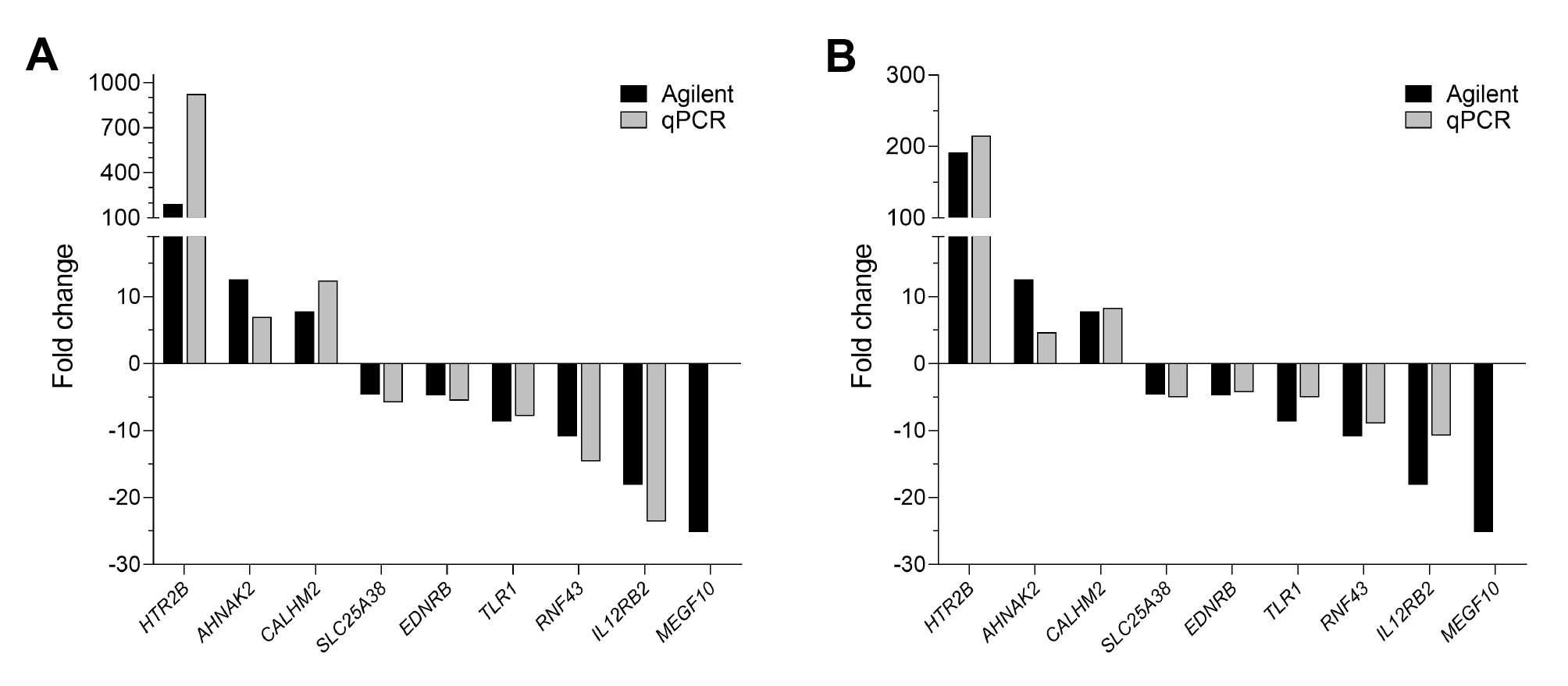

Supplement: Supplementary file 5 — Figure S3. Validation of mRNA expression by qPCR. [file CTM2-13-e1317-s005.jpg]

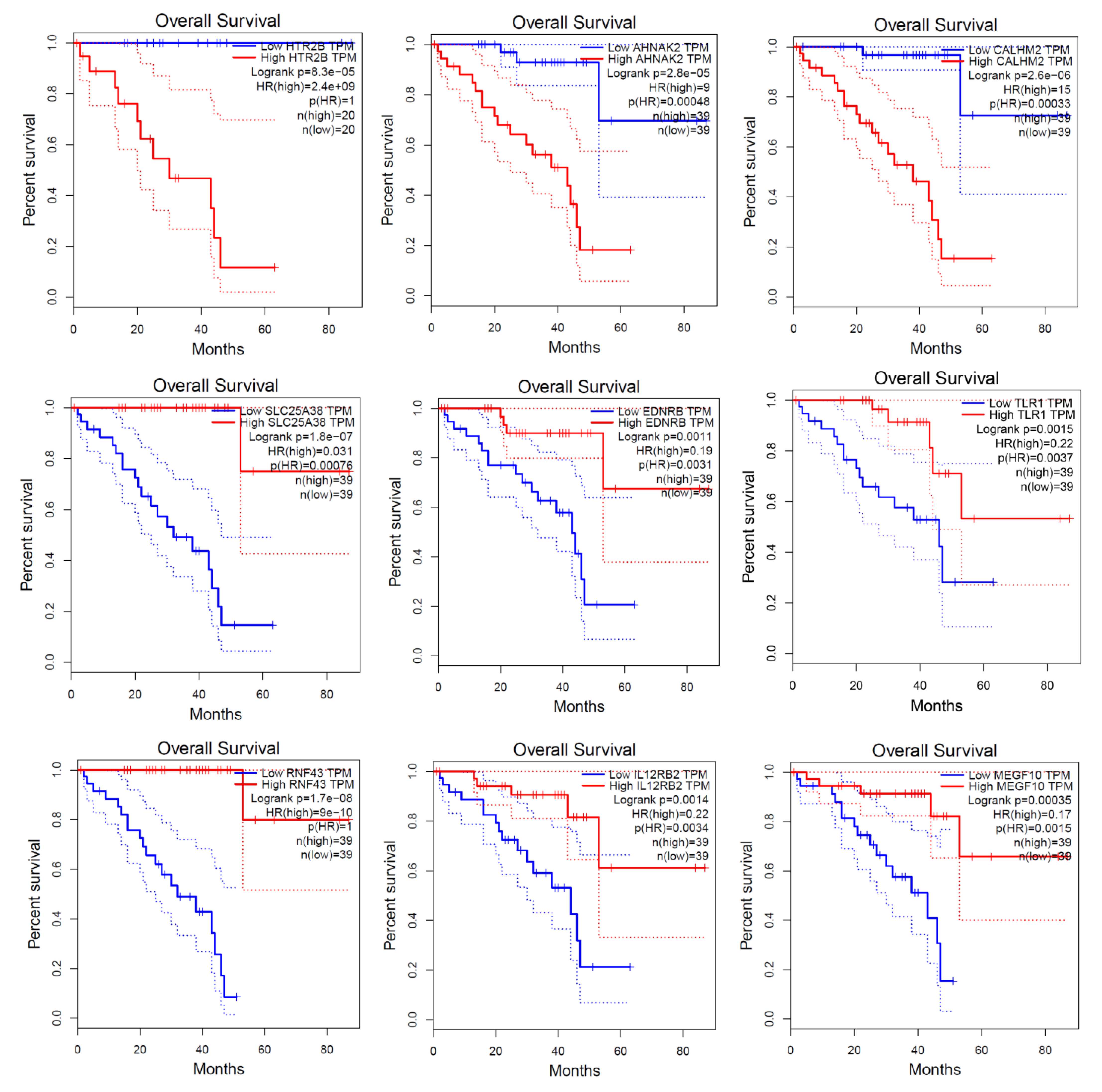

Supplement: Supplementary file 6 — Figure S4. Kaplan‒Meier survival plots constructed based on the gene expression of selected genes from The Cancer Genome Atlas (TCGA) and the Genotype‐Tissue Expression (GTEx) databases using the online Gene Expression Profiling Interactive Analysis (GEPIA) tool. [file CTM2-13-e1317-s002.jpg]
